# Sudden Sensorineural Hearing Loss after Third Dose Booster of COVID-19 Vaccine Administration

**DOI:** 10.3390/diagnostics12092039

**Published:** 2022-08-23

**Authors:** Federica Zoccali, Francesca Cambria, Andrea Colizza, Massimo Ralli, Antonio Greco, Marco de Vincentiis, Carla Petrella, Marco Fiore, Antonio Minni, Christian Barbato

**Affiliations:** 1 Department of Sense Organs, Sapienza University of Rome, 00161 Roma, Italy; 2 Institute of Biochemistry and Cell Biology (IBBC), National Research Council (CNR), Department of Sense Organs, Sapienza University of Rome, Viale del Policlinico 155, 00161 Roma, Italy

**Keywords:** COVID-19, sudden sensorineural hearing loss (SSHL), mRNA vaccine, third administration, booster, otolaryngology

## Abstract

Coronavirus disease 2019 (COVID-19) was declared a pandemic due to its rapid spread worldwide, and its vaccination campaign is considered one of the most historic public hygiene measures in modern medicine. Sudden sensorineural hearing loss (SSHL) is a common emergency that affects patient’s quality of life and requires rapid treatment with steroids. The etiology could be viral or vascular even though in most cases it remains unknown (idiopathic SSHL). During the SARS-CoV-2 vaccination campaign, several rare but serious adverse events have been reported including thrombosis with thrombocytopenia syndrome, myocarditis, and Guillain-Barré Syndrome. ENT adverse events after vaccination were reported too, including cases of sudden sensorineural hearing loss (SSHL), vestibular neuronitis and audio vestibular disorders (such as tinnitus, dizziness, and vertigo). For the first time here, we reported two cases of SSHL after third administration of COVID-19 mRNA vaccine. Even if there is not clear evidence of an association between SSHL and vaccination, adverse effects should be kept in mind since viral infection could be the etiology of SSHL.

## 1. Introduction

Coronavirus disease 2019, caused by severe acute respiratory syndrome coronavirus 2 (SARS-CoV-2), first emerged in late 2019 and due to its rapid spread was declared a pandemic [1]. The pandemic was a massive global health concern which required a rapid development and commercialization of vaccines to limit the spread of the disease and deaths. Although different adverse events following immunization were reported, audio vestibular disorders are an uncommon adverse event, and their risk rates have not increased in conjunction with the COVID-19 vaccinations; there is no reason for any hesitancy towards the vaccination campaign. Furthermore, according to the World Health Organization (WHO), there have been more than 430 million confirmed cases of COVID-19 and nearly 6 million deaths worldwide. As of 21 February 2022, a total of 10,407,359,583 vaccine doses have been administered [1]. During the SARS-CoV-2 vaccination campaign several rare but serious adverse events have been reported including thrombosis with thrombocytopenia syndrome, myocarditis, and Guillain-Barré Syndrome [2]. ENT adverse events after vaccination were reported too, including cases of sudden sensorineural hearing loss (SSHL), vestibular neuronitis, and audio vestibular disorders (such as tinnitus, dizziness, and vertigo) [3]. Sudden sensorineural hearing loss (SSHL) is the rapid onset of hearing loss of at a least 30 dB across three consecutive frequencies occurring within a period of 72 h based on the US National Institute for Deafness and Communication Disorders (NICDC) [4]. Viral infections, vascular compromise, and autoimmune disorders are the most accepted etiopathogenetic mechanisms, even though in 90% of the patients SSHL is characterized as idiopathic due to no cause identification despite investigation [5]. We report the case of two patients with SSHL within five and seven days after COVID-19 vaccination and suppose an association between them. 

## 2. Cases Presentation

### 2.1. Case 1

**Figure 1:**
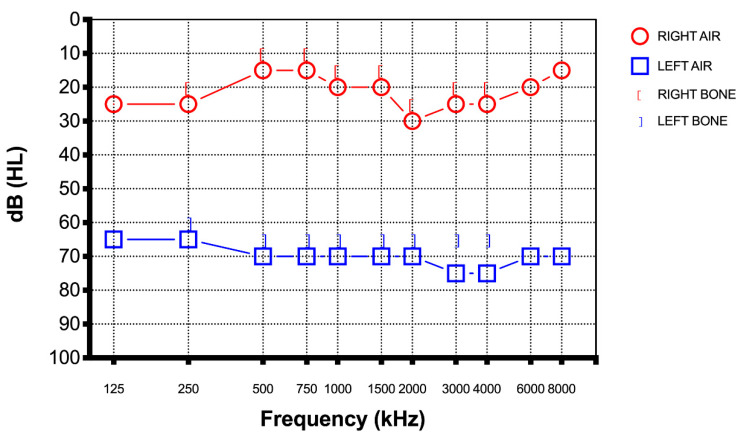
Case 1 pure tone audiometry five days after the third dose of Pfizer-BioNTech COVID-19 vaccine showed profound left SSHL at least 70 dB in every frequency.

A 40-year-old male presented to the emergency department of our hospital with a two-day history of sudden hearing loss of his left side without vertigo, dizziness, and tinnitus five days after the third dose of Pfizer-BioNTech COVID-19 vaccine. He never tested positive for SARS-CoV-2 before this episode. He did not report any history of earache, trauma, or discharge and denied any previous hearing loss. He had a medical history of obstructive sleep apnea syndrome (OSAS), AHI 69.4, which was stable over the past few years. He did not report the use of any medication and he denied use of tobacco and alcohol. There was no relevant personal or family history and he had never previously experienced similar episode. Otoscopy was unremarkable on both ears. A Weber test was lateralized to the right ear while a Rinne test was positive on both sides. Pure tone audiometry was performed and showed profound left SSHL of at least 70 dB in every frequency (250–8000 Hz) (Figure 1).

### 2.2. Case 2

**Figure 2:**
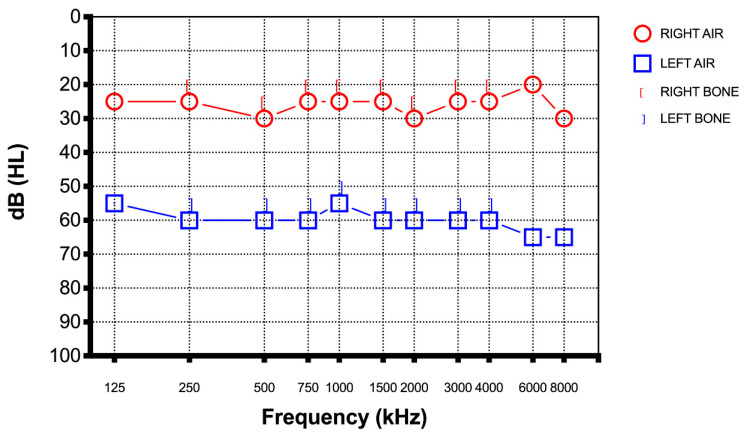
Case 2: pure tone audiometry seven days after the third dose of the Moderna COVID-19 vaccine showed profound right SSHL at least 60 dB in every frequency.

A 67-year-old female presented to our department with a one-day history of sudden hearing loss of the left side seven days after the third dose of the Moderna COVID-19 vaccine. She denied any vertigo, dizziness, or tinnitus, and any previous hearing loss or ear discharge. She tested positive for coronavirus one year before the administration of the third dose. She is a person who experiences allergies and reported the use of an antihistaminic during periods of allergies. She denied use of alcohol or tobacco. There was no relevant personal or family history and she had never previously experienced similar episode. Otoscopy was unremarkable on both ears. A Weber test was lateralized to the right ear while a Rinne test was positive on both sides. Pure tone audiometry was performed and showed profound right SSHL of at least 60 dB in every frequency (250–8000 Hz) (Figure 2).

## 3. Methodology: Investigations, Treatment and Follow-Up

**Figure 3:**
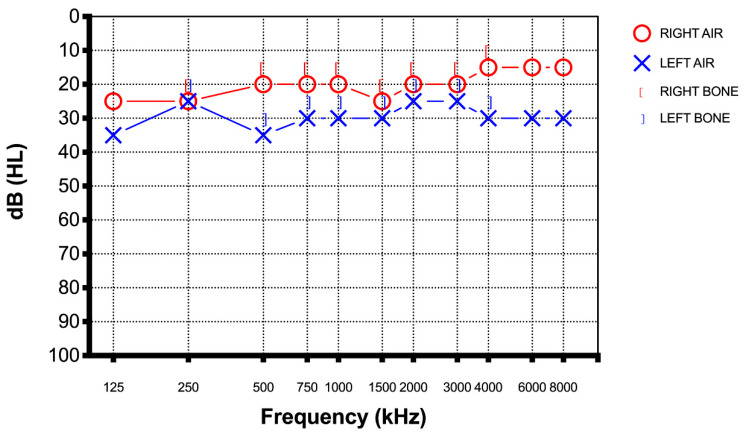
Case 1 pure tone audiometry after therapy showed a total recovery of the hearing loss.

**Figure 4:**
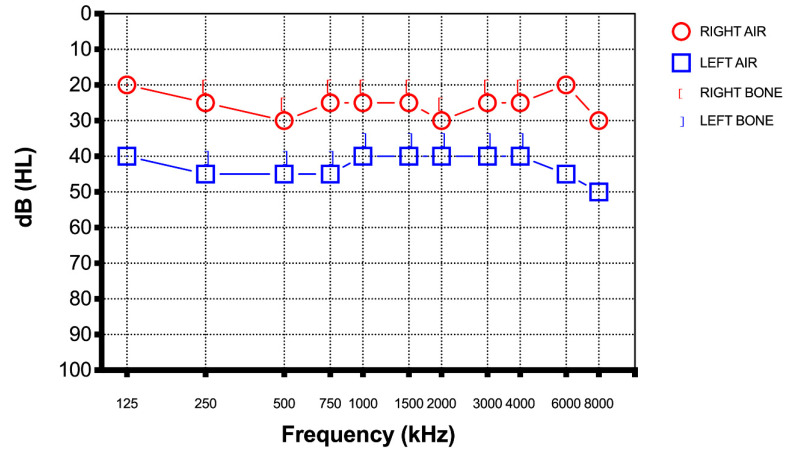
Case 2 pure tone audiometry after therapy showed an almost full recovery of the hearing loss.

On examination, the chest was clear and heart sounds normal, and both were hemodynamically stabile and afebrile. The only symptom they reported was sudden hearing loss in one hear. There were no other neurological deficits. The blood examination was unremarkable, and a chest radiograph and COVID-19 test were negative. A magnetic resonance imaging (MRI) of the brain and internal auditory canal and a magnetic resonance angiography (MRA) were conducted. Both imaging tests in both patients were normal. Due to their recent COVID-19 vaccination, neurologists and internists were asked to assess both patients. Both patients were managed with intratympanic steroid injection (Dexamethasone 5 mg once a day for three alternate days, all repeated for two cycles). This treatment protocol modifies the classic one (three administration in one week). The hearing loss was evaluated every two days with a new pure tone audiometry. After the first week of therapy the audiometry did not reveal any notable improvement. Instead after completing the treatment, based on the intratympanic steroid injection, both patients performed a new pure tone audiogram to our clinic. Both patients reported a significant improvement in their hearing even though the new audiogram showed a total recovery in the patient from Case 1 (Figure 3) and a significant increase of the patient from Case 2’s threshold (almost full recovery) (Figure 4).

## 4. Results and Discussion

Several vaccines have been developed against SARS-CoV-2 in response to the pandemic. The Pfizer-BioNTech consists of messenger RNA (mRNA) encapsulated in a lipid nanoparticle which stimulates innate production of the spike protein, resulting in an immune response producing SARS-CoV-2 specific antibodies [6]. Molecular mimicry between anti-spike COVID-19 antibodies, developed after vaccination, and ear antigens, may explain potential cross-reactivity underlying adverse events. A cross-reactivity between the COVID-19 vaccine and host immune cells may contribute to the development of audio vestibular disorders [6]. Vaccines can cause local or generalized side effects and monitoring for adverse events should continue for years. Otolaryngologic adverse event after COVID-19 vaccination were observed, including several cases of sudden sensorineural hearing loss reported in the Centers for Disease Control and Prevention (CDC) Vaccine Adverse Events Reporting System (VAERS) in the United States [7]. Several cases of SSHL after vaccination have been reported, including after influenza, tetanus, diphtheria, meningococcus, and rabies vaccination. A study by Baxter et al., [8] reported that during the study period >20 million vaccines administered, no evidence of increased risk of immunization compared to matched controls was registered. The etiology of SSHL is usually idiopathic even though it can be ascribed to viral infections, vasculitis, autoimmune disease, and tumor. Instead, the etiology of SSHL after vaccination is still unknown [9]. Formeister et al. reported in a cross-sectional study that there were no associations between the Pfizer-BioNTech or Moderna vaccinations and SSHL [10,11]. Nevertheless, viral antigens after vaccination could induce an immunologic and inflammatory responses resulting in a release of antibodies and cytokines which could cause autoimmune response directing antibodies to the cochlea. All of those mechanisms might result in vasculitis and vascular ischemia of the cochlea [12]. However, these etiologies are suspected to be causes of SSHL regardless of vaccination. 

On the other hand, in a recent large Israel population-based cohort study by Yanir and collaborators, was suggested that the BNT162b2 mRNA COVID-19 first and after the second vaccine dose might be associated with increased risk of SSHL [13]. Conversely, sudden sensorineural hearing loss within a few days after COVID-19 vaccination demonstrates the possibility that vaccination is a significant cause due to the booster effect that stimulates and activates the immune system earlier. In addition, the labyrinthine artery is a terminal artery which mainly supplied cochlea. In fact, cochlea is very sensitive to circulatory alterations and ischemia. Thrombosis or vasospasm of the internal auditory artery are one of the main causes surrounding idiopathic sudden sensorineural hearing loss. Safety concerns about increased risk for thrombotic events have led to a temporary halt of vaccination with the Oxford-AstraZeneca COVID-19 vaccine [6]. It was observed that the percentage of deaths following thrombosis is very low and it suggested that the advantage of immunization was greater than the risks of its potential side effects. Moderna is an mRNA vaccine which allows the mRNA for spike proteins to enter the host cells, increasing an intense immune response. The mRNA vaccines could elicit an autoimmune reaction resulting in type I interferon production, and a cross-reactivity between anti-spike SARS-CoV-2 antibodies and ear antigen is a possibility that could link COVID-19 vaccines to audio vestibular disorders [7]. The treatment of SSHL after COVID-19 vaccination is the same as in the absence of the vaccine. Good clinical practice guidelines indicated that corticosteroids can be administered within two weeks as initial therapy for SSHL [14]. Corticosteroids, immunosuppressive agents, or diseases of vaccine recipients can affect the antibody response to the vaccination. Intratympanic steroid injection rather than systemic steroid injection can be considered to prevent an insufficient immunization after vaccination, as it might not suppress the immune system systemically. In our opinion, a therapy protocol based on intratympanic steroid injection (Dexamethasone 5 mg once a day for three alternate days, all repeated for two cycles) could be useful as initial treatment of SSHL. Although this treatment protocol modifies the classic one (three administration in one week), our clinical experience in the treatment of post COVID-19 hearing loss supported us in using this therapy with good results. If a systemic high dose of steroid was administered during the vaccination period, a measurement of the antibody titer could be necessary after completion of the vaccination [14].

## 5. Conclusions

The incidence of SSHL is between 5 and 30 cases per 100,000. Wichova et al. affirmed that SSHL increased among their patients from 2019 and 2021, suggesting an association between COVID-19 or COVID-19 vaccination and SSHL [15]. The opposite risk of hearing loss after COVID-19 vaccination was commented by Slomski [16]. Although SSHL can occur in a few individuals after COVID-19 vaccinations, the etiologic mechanism is still unclear [17,18]. For future research, we suggest exploring modifications of potential biomarkers associated with SSHL in order to elucidate potential biological mechanisms bridging sensory loss and COVID-19 mRNA vaccination. We believe that this could be an interesting field of research and development. Our cases demonstrate a temporal association between COVID-19 vaccination and sudden sensorineural hearing loss. In fact it should be carefully evaluated and not be dismissed as a coincidental event [19]. As in SSHL unrelated to vaccination, a quick systemic or intratympanic steroid administration is required in SSHL after COVID-19 vaccination [20]. In conclusion, this adverse event should be kept in mind and rapidly treated although it should not prohibit patients from accessing COVID-19 vaccination.

For future research, we suggest exploring modifications of potential biomarkers associated with SSHL that contribute to elucidating the potential biological mechanisms bridging sensory loss and COVID-19 mRNA vaccination.

## Data Availability

Not applicable.
